# Preparation and Performance Characterization of a Composite Film Based on Corn Starch, κ-Carrageenan, and Ethanol Extract of Onion Skin

**DOI:** 10.3390/polym14152986

**Published:** 2022-07-23

**Authors:** Cuntang Wang, Yueyi Lu, Ziyu Li, Xuanzhe An, Zengming Gao, Shengxin Tian

**Affiliations:** 1 College of Food and Bioengineering, Qiqihar University, Qiqihar 161006, China; lu18945341162@163.com (Y.L.); lonelee96@163.com (Z.L.); an376443653@163.com (X.A.); gao1513868@163.com (Z.G.); 18600627806@163.com (S.T.); 2 College of Food Science, Northeast Agricultural University, Harbin 150030, China

**Keywords:** corn starch, κ-carrageenan, ethanol extract of onion skin, active film, physicochemical properties, biological activity

## Abstract

Using corn starch (CS) and κ-carrageenan(κC) as the raw material and active composite, respectively, films containing different concentrations of ethanol extract of onion skin were prepared. The effects of different concentrations of ethanol extract of onion skin (EEOS) on the physicochemical properties, as well as the antioxidant and antibacterial properties, of CS/κC films were also discussed. The addition of ethanol extract of onion skin inhibited the recrystallization of starch molecules in the composite films. It affected the microstructure of the composite films. The color of the composite films was deepened, the brightness was reduced, and the opacity was increased. Water vapor permeability increased, tensile strength decreased, and elongation at the break increased. The glass-transition temperature decreased. The clearance of DPPH radicals and ABTS cation radicals increased. Moreover, when the concentration of EEOS was 3%, the antioxidant effect of the films on oil was greatly improved and could effectively inhibit *Staphylococcus aureus* and *Escherichia coli*. The above results showed that adding ethanol extract of onion skin improved the physicochemical properties and biological activities of the CS/κC composite films, so CS/κC/EEOS composite films can be used as an active packaging material to extend food shelf-life. These results can provide a theoretical basis for the production and application of corn starch/κ-carrageenan/ethanol extract of onion skin composite films.

## 1. Introduction

Traditional petroleum-based plastic films are widely used as packaging materials for their low-density, low-cost, and good molding properties [1]. However, the slow biodegradation of food plastic packaging materials has caused global increases in plastic waste [2]. Thus, the development of eco-friendly and safe packaging materials has emerged as an area of interest [3]. Concerns about environmental issues associated with the large disposal of nondegradable plastics and diminishing supplies from oil sources drive efforts to investigate sustainable materials that are degradable and are from renewable sources [4]. When considering the sustainability of the ecosystem, more people prefer to transfer their interest to degradable materials [5]. Biopolymer materials have attracted more and more attention due to their excellent properties, such as biodegradability, safety, biocompatibility, and renewability [6]. In recent years, biological materials such as polysaccharides, proteins, and lipids have been highly focused on [7]. Biodegradable films can be developed using proteins, carbohydrates, lipids, or compounds [2]. Moreover, films based on biodegradable materials are often incorporated with other bioactive compound blends, such as plant extracts, to satisfy the changing preferences and expectations of modern consumers [8]. Among natural and renewable resources, starch, as a significant raw material, has attracted extensive attention due to its low production cost, availability, and biodegradability, as well as its odorless, colorless, and nontoxic biodegradable film qualities and its ease of use in film production [9]. Among biopolymer materials, corn starch is considered one of the most promising plastic alternatives since it is abundant, inexpensive, biodegradable, edible, and presents good film-forming ability [10]. Although starch-based materials have good oxygen barrier properties, compared with traditional plastics, the use of corn starch to produce biodegradable plastics has disadvantages, such as poor moisture resistance and poor mechanical properties, and its application is limited [2]. To overcome these shortcomings, mixing starch with other natural biopolymers to form composites has received much attention [11].

Carrageenan is one of the algal colloids among several polysaccharides extracted from seaweed. It consists of alternating disaccharide units of β-(1-4)-3,6-anhydrous-D-galactose and α-(1-3)-D-galactose. It is commercially classified into three categories, κ (kappa), ί (iota), and λ (lambda), according to differences in the content of the sulfate group and its position in the structure [12]. Carrageenan is increasingly regarded as a promising renewable biomaterial with excellent film-forming ability and has great potential as a substitute for traditional synthetic plastics. In recent decades, Carrageenan has been widely used in edible films and coatings [13]. However, carrageenan films have limitations. The κ-carrageenan monomer selected by the research institute has only one sulfate group and has good film-forming properties but poor mechanical properties. In this experiment, corn starch was mixed with κ -carrageenan and could form a film with good mechanical properties [12]. To expand the scope of application, a large number of studies have been carried out on how to improve the performances of films [12]. One way to improve the performance of thin films is to add active compounds. Natural compounds are preferred instead of synthetic additives. Natural extracts rich in polyphenols are an effective way to prevent lipid oxidation in food and can be used as a functional additive. Some studies on biopolymer films based on natural antioxidants have been reported [14]. Due to their superior *biodegradable*, *biocompatible*, and economic advantages, composite films mixed with natural extract materials have been widely studied and applied [13].

The onion (an *Allium of the Liliaceae* family) is one of the most essential and widely eaten vegetables globally. It is the second most cultivated crop after tomatoes. However, about 500,000 tons of inedible onion waste are produced every year [15]. Onion waste is mainly made up of onion skins, which are rich in bioactive phenolic compounds. Polyphenols are a kind of natural antioxidant, and their groups include flavonoids and anthocyanins [16]. Purple onions obtain their color from anthocyanins in their epidermal cells. In flavonoids, anthocyanins are considered the most biologically active compounds with a high antioxidant performance [17]. Although onion skins are rich in fiber and flavonoids, the dry, outer layer of onion skin is considered waste and is discarded, resulting in a significant loss of phenolic compounds [18]. Phenolic compounds can act as free radical scavengers and metal ion chelators to inhibit lipid oxidation [16]. Therefore, onion skin extract can be added to films as antioxidant material to prepare food-active packaging and to develop a biodegradable antioxidant film.

However, there are few reports on preparing composite films by blending onion skin extract with corn starch and κ-carrageenan. In this study, ethanol extract of onion skin are added to a composite film to improve its physical and chemical properties. The effects of different contents of EEOS on the properties of CS/κC films are also evaluated to select a good food-packaging film. The characterization of CS/κC/EEOS composite films investigates the effects of EEOS on film properties, including Fourier-transform infrared spectroscopy, crystal structure, morphology, optical properties, mechanical properties, water vapor permeability, thermodynamic property, antioxidant activity, and antibacterial activity. We hope to provide environment-friendly film materials for food packaging. Therefore, the purpose of this study is to prepare environmental-friendly and functional CS/κC/EEOS composite films with different concentrations of ethanol extract of onion skin as raw materials.

## 2. Materials and Methods

### 2.1. Materials and Reagents

Purple onion skin was obtained from the Liu yuan Market (Qiqihar, China); corn starch, was obtained from Heilongjiang YuFeng Corn Development Co., Ltd. (Heilongjiang, China); food-grade κ-carrageenan was obtained from Qingdao DeHui Marine Biotechnology Co., Ltd. (Qingdao, China); beef paste and tryptone (biochemical reagent) were obtained from Beijing AoBoxing Biotechnology Co., Ltd. (Beijing, China); *Staphylococcus aureus* (ATCC29213) and *Escherichia coli* (ATCC25922) were obtained from the College of Food and Bioengineering of Qiqihar University (Qiqihar, China); and 2,2-Diphenyl-1-picrylhydrazyl (DPPH) and 2,2′-azino-bis-(3-ethylbenzothiazoline-6-sulfonic acid) (ABTS) were obtained from Sigma-Aldrich Chemical Co. (St. Louis, MO, USA). All other chemical reagents (analytically pure) were obtained from Tianjin KaiTong Chemical Reagents Co., Ltd. (Tianjin, China).

### 2.2. Preparation of Ethanol Extract of Onion Skin

The purple onion skins were washed, blasted in an air oven (GZX-9146MBE, Shanghai BoXun Industrial Co., Ltd., Shanghai, China) to dry them, crushed with a shredder (GX-220, Zhejiang GaoXin Industrial and Trade Co., Ltd., Yongkang, China), and sifted. A specific mass of purple onion skin powder was weighed, and ethanol solution with the 70% volume fraction was added in a ratio of 1:2 (W:V). The solution was extracted at 25 °C for 2 h, then the leached liquor was centrifuged at 6000 r/min for 10 min, and the supernatant was obtained for standby application. After centrifugation, the sediment was extracted and centrifuged again. Two centrifugates of supernatants were combined. The supernatant was transferred to a rotary evaporator (2L-ARE, Shanghai HaoZhuang Instrument Co., Ltd., Shanghai, China) and concentrated at 50 °C. Then, the concentrated solution was freeze-dried with a vacuum freeze-dryer (2.5 L freezer dryer, Labconco company, Kansas City, MO, USA) to obtain the EEOS. The EEOS was stored in a refrigerator at −20 °C until ready for use.

### 2.3. Preparation of CS/κC/EEOS Complex Films

A certain amount of corn starch was added into distilled water to prepare a suspension of corn starch with a concentration of 3% and was gelatinized at 90 °C for 30 min. An amount of 30% κ-carrageenan (W/W: κ-carrageenan mass/starch dry-base mass) was added, and we continued to heat and stir at 90 °C for 30 min. Leaving one control, amounts of 1%, 3%, and 5% ethanol extract of purple onion skin (W/W: extract weight/starch dry-base weight) were added and, respectively, called CS/κC films, CS/κC/EEOS Ⅰ films, CS/κC/EEOS Ⅱ films, and CS/κC/EEOS Ⅲ films. We continued to stir for 30 min, added 25% glycerin (W/W: glycerin mass/starch dry-base mass), and continued to heat and stir at 90 °C for 30 min, using hot water to complement the evaporated water. The solution was ultrasonicated at 80 °C for 30 min to remove bubbles, and the composite film solutions were prepared. A polyethylene ring with a diameter of 120 mm was fixed on a glass plate covered with release paper. An amount of 25 g of film solution was cast in the polyethylene ring and fixed for 15 min. The samples were placed in an air-blast drying oven and dried at 40 °C for 12 h. Then, the films were uncovered. The films were placed in a dryer at 25 °C with a relative humidity of 56.8% (NaBr saturated solution) and were balanced for 72 h to determine the parameters of the films.

### 2.4. Characterization

#### 2.4.1. Determination of Fourier-Transform Infrared Spectroscopy (FTIR)

Infrared spectra were measured with a Fourier-transform infrared spectrometer (Spectrum-100, PerkinElmer company, Waltham, MA, USA). After being balanced and dried, the films were placed on the ATR accessory. The test temperature was 25 °C, the wavenumber was 4000–650 cm^−1^, the resolution was 4 cm^−1^, and the number of scans was 32.

#### 2.4.2. X-ray Diffraction Analysis (XRD)

The samples were analyzed with an XRD instrument (Rint-2000, Neology Co., Ltd., Tokyo, Japan). A Cu target and a graphite monochromator were used. The test parameters were as follows: the voltage was 45 kV, the current was 20 mA, the scanning range was 5~80° (2θ), and scanning rate was 2 (°)/min.

### 2.5. Morphology and Optical Properties

#### 2.5.1. Scanning Electron Microscope (SEM)

Each composite film sample was cut into a brittle rectangle of 4 cm × 6 cm and was broken using liquid nitrogen. A scanning electron microscope (S-4300, Hitachi, Japan) was used for scanning the microstructure. The scanning voltage was 2.00 KV, and the current was 64.0 μA. The surface and cross-section structures of the films were photographed and observed.

#### 2.5.2. Optical Properties

For the measure of color and luster, the a * value (red and green), b * value (yellow and blue), and L * value (brightness) of the film samples were measured with a visible spectrophotometer (UPG-722, Beijing YouPu General Technology Co., Ltd., Beijing, China). Before measurement, it was calibrated with a standard board (a * = −5.80, b * = 9.25, and L * = 103.98). Each sample was measured three times, and the results were averaged.

For the measure of opaqueness, according to Sukhija et al. [19], each film sample was cut into a 10 mm × 45 mm rectangle, which was closely attached to the inner wall of an empty colorimeter. The empty colorimeter was used as a reference, and the absorbance values of the samples were measured at 600 nm. The opacity was calculated according to Equation (1). Each sample was measured three times, and the average value was taken.

(1) $$\mathrm{Opaqueness}=\frac{A_{600}}{X}$$

In the formula, A_600_ is the absorbance value at a wavelength of 600 nm, and X is the film thickness in mm.

For appearance, the composite film was covered on A4 white paper, and an image was captured of the composite film.

### 2.6. Mechanical and Barrier Properties

#### 2.6.1. Determination of Mechanical Properties

The mechanical properties of the films were slightly modified by referring to Mu et al. [20]. The films were cut into 6 cm × 2 cm rectangles, the test speed was 2 mm/s, and the initial clamping distance was 20 mm. The tensile strength (MPa) and elongation at the break (%) of the film samples were measured with a texture tester (TA. XT plus, Stable Micro System company, Surrey, UK). Each sample was measured three times, and the results were averaged. The mechanical properties of the carrageenan films were calculated according to Equations (2) and (3):

(2) $$\mathrm{TS}=\frac{F_{\max}}{S}$$

In the formula, TS is the tensile strength in MPa, F_max_ is the maximum load when the film breaks in N, and S is the film cross-sectional area in mm^2^.

(3) $$\mathrm{EB}\%=\frac{L_{1}-L_{0}}{L_{0}}\times 100\%$$

In the formula, EB is the elongation at the break as a percentage, L_1_ is the length of the film after stretching in mm, and L_0_ is the initial length of the film at 20 mm.

#### 2.6.2. Determination of Water Vapor Transmittance (WVP)

Ten grams of anhydrous CaCl_2_ was placed in a blast-drying oven, dried at 110 °C for 2 h, and placed in a 35 mm × 90 mm weighing bottle. The prepared film sample was covered over the weighing bottle’s mouth, sealed, and placed in a dryer with distilled water at the bottom. The weight of the measuring bottle was measured every 24 h and continuously for 10 days. Each sample was measured three times, and the results were averaged. The water vapor permeability of the film could be calculated according to Equation (4):

(4) $$WVP=\frac{W}{t\times A}\times \frac{X}{∆P}$$

In the formula, *WVP* is the water vapor permeability (g·mm/m^2^·d·kPa), W is the the total weight of the measuring bottle after film sealing (g), t is the time (d), A is the permeable film area (m^2^), X is the film thickness (mm), and ΔP is the vapor pressure difference between the two sides of the film (1.583 kPa).

### 2.7. Differential Scanning Calorimetry (DSC)

Differential scanning calorimetry was used for the thermal stability analysis. About 4 mg of film samples dried with a dryer were taken and sealed in an aluminum crucible, using an empty aluminum crucible as a reference. Nitrogen was the protective gas, with a flow rate of 20 mL/min. The heating rate was 10 °C/min, and differential scanning calorimetry was performed in the temperature range from 20 °C to 250 °C.

### 2.8. Determination of Total Phenol Content (TPC)

The total phenol content of the composite film was determined using the Folin-phenol colorimetric method. A total of 125 mg film sample was immersed in 15 mL distilled water for 24 h to obtain a film immersion solution. Amounts of 0.1 mL film immersion solution, 7 mL distilled water, and 0.5 mL Folin-Phenol were successively added to a 50 mL conical flask and gently shaken. After 8 min of rest, 1.5 mL 10 wt% sodium carbonate solution and 0.9 mL of distilled water were added successively, and the composite solution was put into a dark room to avoid light for 2 h. Then, the absorbance of the mixture was measured at 765 nm with an ultraviolet spectrophotometer. A series of aqueous gallic acid solutions with concentrations between 0 and 15 μg/mL were prepared, and the absorbance at 765 nm was measured according to the above steps. The standard curve was drawn with the concentration of aqueous gallic acid as the abscissa and the absorbance at 765 nm as the ordinate. The equation y = 0.117x + 0.0171 (R^2^ = 0.9995) was obtained. According to this equation, the total phenol content was calculated using the absorbance of the composite film immersion solution at 765 nm. The total phenolic content of the sample was expressed as the mg gallic acid equivalent (GAE) per gram of dry matter (DW), or GAE mg/DW g. Each sample was measured three times, and the results were averaged.

### 2.9. Antioxidant Properties

#### 2.9.1. Determination of Antioxidant Capacity In Vitro

A total of 10 mg film sample was mixed with a methanol DPPH solution (0.2 mM; 1.5 mL). The mixture was put into a dark chamber for reaction and kept away from light for 30 min at indoor temperature. Then, the absorbance of the mixture was measured at 517 nm with a UV-vis spectrophotometer, using the DPPH methanol solution as the control [21]. Each sample was measured three times, and the results were averaged. The equation used for calculating the free radical clearance rate (Equation (5)) was as follows:

(5) $$k=\frac{A_{C}-A_{s}}{A_{C}}\times 100\%$$

In the formula, *k* is the free-radical-scavenging ability (%), A_C_ is the the absorbance of control group, and A_S_ is the absorbance of sample

A total of 10 mg film sample was mixed with ABTS free radical working solution and kept from light at room temperature for 6 min. Using a UV-vis spectrophotometer, the absorbance of the mixture was determined at 734 nm. An acetic acid buffer solution was used instead of an extract sample solution as a blank control. Each sample was measured three times, and the results were averaged. The free radical clearance rate was calculated according to Equation (5).

#### 2.9.2. Determination of Peroxide Value (POV)

An amount of 5 g solid lard was wrapped in the film, and the film was heat-sealed and placed in a blast-drying oven at 60 °C to accelerate oxidation. The samples were sampled at every 24 h interval and measured continuously for 7 d. The POV value was determined according to the method of Gao et al. [22]. Each sample was measured three times, and the results were averaged.

### 2.10. Determination of Antibacterial Properties

Cultured *Escherichia coli* and *Staphylococcus aureus* strains were diluted ten times with sterile normal saline as the initial bacterial solution. The thin-film samples were cut into discs with diameters of 7 mm with a sampler. After being sterilized with ultraviolet radiation, they were placed in a culture medium containing 0.1 mL target bacteria solution and incubated in a constant-temperature incubator at 37 ± 1 °C for 24 h. Use vernier calipers to measure the diameters of the membrane inhibition zones, the antibacterial activity of the film was judged by the size of the antibacterial area. Each sample was measured three times, and the results were averaged.

### 2.11. Data Processing

The experimental results were expressed as average values ± standard deviation. Duncan’s multiple range test in SPSS 26 software (SPSS Inc., Chicago, IL, USA) was used to analyze the significance of the difference, and Origin 2019 software (Microsoft, WSU, USA) was used to plot.

## 3. Results

### 3.1. FTIR

**Figure 1:**
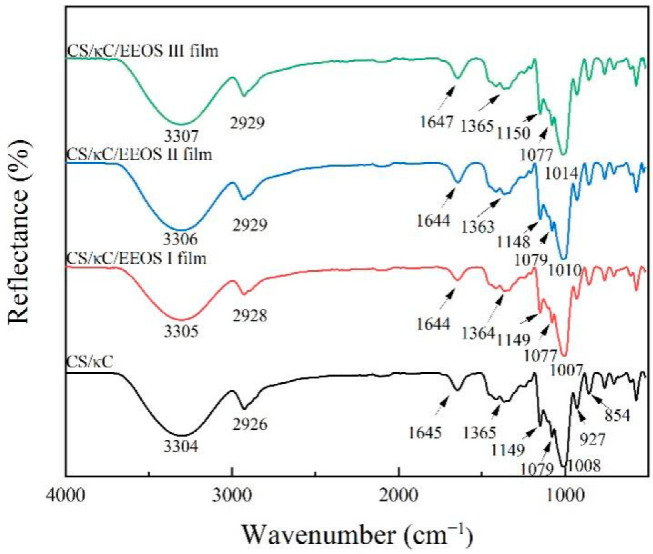
FTIR spectra of CS/кC, CS/кC/EEOS I, CS/кC/EEOS II, and CS/кC/EEOS III composite films.

The infrared spectra refer to the absorption bands formed by molecules selectively absorbing specific frequencies under infrared irradiation. FTIR spectroscopy can not only measure the functional groups of each component in the film but also can be used to detect the molecular interactions between the components of the film [23]. The interactions between EEOS and the CS/κC composite films were further analyzed by Fourier-transform infrared spectroscopy (FTIR). The FTIR spectra of CS/κC/EEOS composite films in the range of 4000~520 cm^−1^ are shown in Figure 1.

For the CS/κC films, the starch contained many hydroxyl groups. There was a wide band at 3304 cm^−1^, where the peak appeared as O-H vibration stretching, and the shifting of the O-H stretching peak suggested interaction between the polymer and additives via H-bonding [24]; the peak at 2926 cm^−1^ was a C-H stretching vibration peak. The absorption peak at 1645 cm^−1^ was attributed to the tight binding between starch and water. Meanwhile, it was shown that the characteristic peak is related to the crystallinity of starch, and the decrease in crystallinity leads to an increase in the strength of the characteristic peak [25]. The C-H and C-O stretching of starch were respectively observed at 1365 cm^−1^ and 1149 cm^−1^. Glucose pyranose ring vibration was seen at 993–1079 cm^−1^. For carrageenan, the stretching vibration peak of the O-H bond appeared at 3304 cm^−1^. The stretching vibration peak of the C-H bond occurred at 2926 cm^−1^. The stretching vibration peak of the C=O bond appeared at 1645 cm^−1^. The peak at 1365 cm^−1^ was from the in-plane-bending vibration of the O-H bond. The absorption peak at 1008 cm^−1^ came from the stretching vibration of the C-O bond. The characteristic peaks at 1149, 1079, 927, and 854 cm^−1^, respectively, corresponded to the sulfate; glycosidic bond; 3, 6-anhydrous galactose; and galactose 4-sulfuric acid in the κ-carrageenan structure [12]. 

After adding EEOS, the infrared spectra curves of the composite films did not change significantly, indicating that the addition of EEOS did not cause structure change in the composite films and that there was good compatibility between the two. The absorption peaks at 2926 and 1008 cm^−1^, respectively, moved to 2929 and 1014 cm^−1^, which may be due to the increase in chemical bond force and stretching vibration, making the absorption peak move to a higher wavenumber. Similar changes in band location and peak strength were observed when food anthocyanin was added to starch/polyvinyl alcohol films [26]. These results indicated that the interactions between components can be identified with FTIR. The increase in band strength at 3304, 2926, and 1008 cm^−1^ of the composite films was caused by hydrogen bonding between the composite films and EEOS [27]. 

### 3.2. XRD Analysis

**Figure 2:**
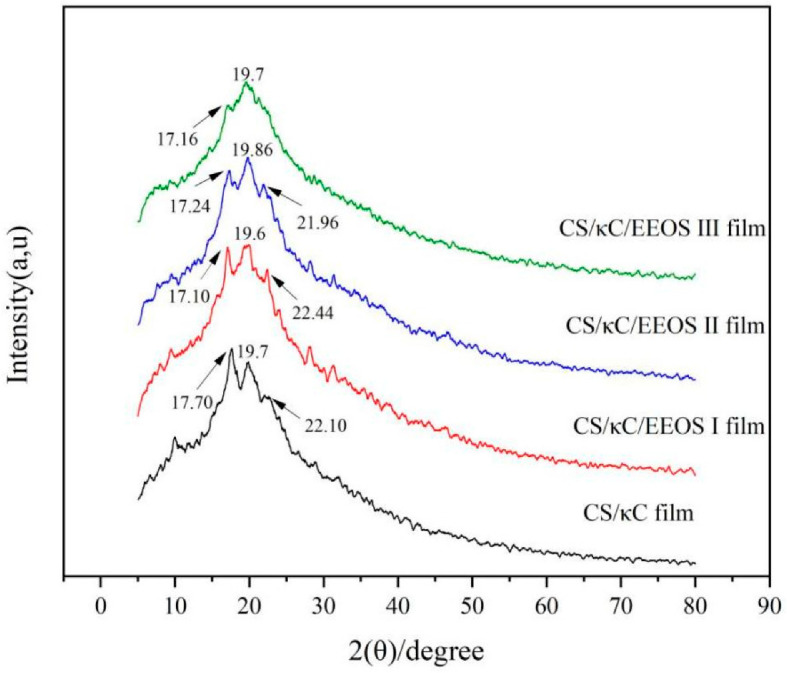
XRD spectra of CS/кC, CS/кC/EEOS I, CS/кC/EEOS II, and CS/кC/EEOS III composite films.

The crystallinity of polymers can be analyzed with XRD. The X-ray diffraction patterns of a CS/κC/EEOS composite film are shown in Figure 2.

CS/κC films had three diffraction peaks at 2θ = 17.7°, 2θ = 19.7°, and 2θ = 22.1°. The diffraction peaks at 2θ = 17.7° and 19.7 were due to the recrystallization of amylose and amylopectin during the storage of the starch composite films [28]. After adding the EEOS, the diffraction peak at 2θ = 17.7° decreased with the addition of EEOS. The diffraction peak at 2θ = 22.1° increased with the addition of EEOS. However, when the amount of EEOS added reached 5%, the diffraction peak here disappeared, and the composite films only showed an obvious peak at 19.7°. The addition of EEOS affected the crystal structure of the composite film, which may be because of the interaction between the polyphenols contained in EEOS and the starch molecules, affecting the order of the starch molecular structure and preventing the recrystallization of starch. This is similar to the research results of Ren [11]. The crystallinity of the starch film comes from the crystallization of amylose and amylopectin. The crystallization rate of amylopectin is slow and occurs during storage, so the recrystallization of starch composite films occurs during storage. The molecular interaction between EEOS and the CS/κC matrix affects the recrystallization of the starch. Sun et al. made a similar discovery, finding that the interaction between anthocyanins and polymers and the promotion of the spatial reconfiguration of polymer chains affected the lattice structure of potassium chloride [12]. Interaction between polymers causes the fine dispersion of bulky polyphenolic compounds, which physically prevents close contact of the polymers and prevents crystallization [29]. The results showed that the addition of plant extracts had a good effect on maintaining the good mechanical properties of the composite films during storage.

### 3.3. SEM

**Figure 3:**
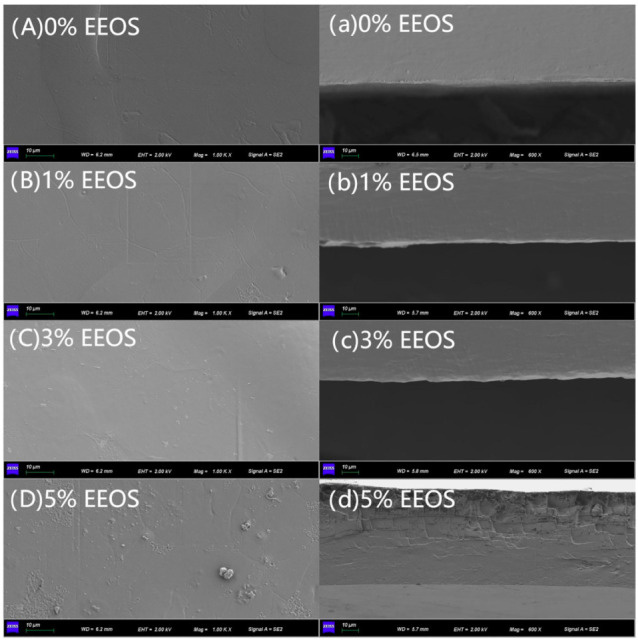
SEM micrographs of the surfaces and cross-sections of composite films. (**A**–**D**) are the surfaces, and (**a**–**d**) are the cross-sections of composite films.

SEM can observe the microstructure of the films and the interactions and influences between the films and additives. The surface and cross-section microstructures of the CS/κC/EEOS composite films are shown in Figure 3. 

As can be seen from Figure 3A–D, the CS/κC films without EEOS were relatively compact and smooth, indicating that there was good compatibility between corn starch and carrageenan. With the increase in EEOS addition from 1% to 5%, the surfaces of the composite films gradually presented a convex structure, and the presence of insoluble particles could be observed, which indicated that a small amount of EEOS was insoluble in the films, making the film surfaces uneven. Gasti et al. [30] also observed a similar phenomenon. It can be seen from Figure 3a–d that the cross-sections of the films were identical to the surface structure. The CS/κC film had a more compact structure and a smooth cross-section. With the addition of EEOS, the section began to gradually become rough. When the content of EEOS reached 5%, the cross-sections of the films became coarser, which corresponded to the phenomenon of the surface structure. These results showed that EEOS was evenly distributed at low concentrations, but the effect of uniformity was more significant at higher concentrations. Due to the higher extract concentration, the polymer network was destroyed, and the structure was coarser and less compact. Furthermore, the spatial structure of the composite film was affected. Similarly, reports of novel films combining starch with polyvinyl alcohol and food anthocyanin have also shown that a low content of PSPE in films had a compact structure. In contrast, excessive PSPE had a greater impact on the uniformity of the films and showed a rough surface [26].

### 3.4. Optical Properties

Color is an important index to measure food-packaging films. a *, b *, and L * are used to distinguish color differences. A positive a * value represents the redness value of a sample, and a positive b * value represents the yellowness value of a sample [26]. CS/κC films containing different concentrations of EEOS were prepared. The optical properties of the CS/κC/EEOS composite films are shown in Table 1.

For the CS/κC/EEOS films, the a * value increased from −1.22 ± 0.04 to 17.26 ± 0.23 (*p* < 0.05); the b * value increased from −0.18 ± 0.08 to 18.06 ± 0.85 (*p* < 0.05) and showed a significant increasing trend (*p* < 0.05). It indicated that the redness value and yellowness value of the composite films increased. L * decreased from 90.24 ± 0.33 to 59.88 ± 0.91 (*p* > 0.05), and the opacity of the composite films increased significantly, indicating that the color of the composite films gradually deepened, while the brightness and opacity gradually decreased. The color of the composite films gradually changed from colorless and transparent to red and yellow. With the increase in EEOS concentration, the color of the films gradually deepened, which was consistent with the images of the films. The color change in the CS/κC film may be caused by EEOS. Many studies have reported color changes in biopolymer films containing plant extracts, such as chitosan films containing purple tomato anthocyanin [31], chitosan/PVA/ZnO films containing purple potato or rose anthocyanins [7], and cassava starch films containing lycium berry anthocyanins [27].

### 3.5. Analysis of Mechanical Properties

**Figure 4:**
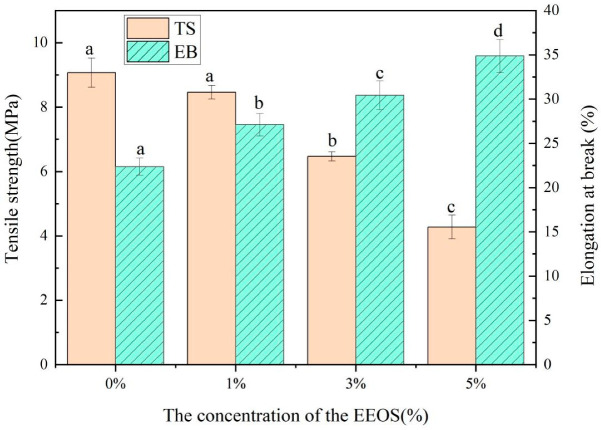
Tensile strength and elongation at the break of the CS/κC, CS/кC/EEOS I, CS/кC/EEOS II, and CS/кC/EEOS III composite films. ^a–d^ Values are given as means ± standard deviation. Different letters in the same line indicate significant difference (*p* < 0.05).

The mechanical properties of composite films are expressed by tensile strength and elongation at the break. Tensile strength (TS) and elongation at the break (EB) are respectively used to reflect the mechanical resistance and flexibility of food packaging. The mechanical properties of the CS/κC/EEOS composite films are shown in Figure 4.

When the content of EEOS was 0–5%, the tensile strength of the films decreased from 9.07 to 4.27 MPa, and the elongation at the break increased from 22.37% to 34.87% with the increase in the content of EEOS. The additive amount of EEOS affected the TS and EB (*p* < 0.05). With the rise in the EEOS addition level, TS showed a downward trend, which may be due to the damage to the polymer network caused by excessive anthocyanins in the extract, resulting in a reduction in TS [26]. At the same time, all the films added with EEOS showed greater EB than the CS/κC films. EB showed an opposite trend to TS. The decrease in TS in concurrence with the increase in EB suggested increasing flexibility of the films due to plasticization effects [32]. EEOS could change the mechanical properties of the films, which may be caused by the molecular interaction between anthocyanins in the extract and the film-forming matrix [33]. Sun et al. found a similar decrease in TS and an increase in EB in κC/HMx/Pmy membranes, which they attributed to the formation of new hydrogen bonds between anthocyanins and polymers. The results coincided with the FITR and XRD analyses [12].

### 3.6. WVP

**Figure 5:**
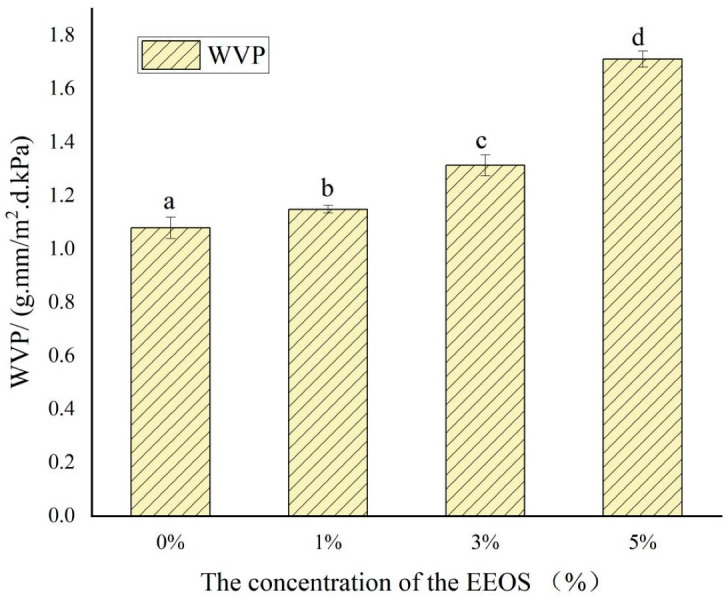
WVP of CS/κC, CS/кC/EEOS I, CS/кC/EEOS II, and CS/кC/EEOS III composite films. ^a–d^ Values are given as means ± standard deviation. Different letters in the same line indicate significant difference (*p* < 0.05).

Due to the role of water in metamorphic reactions, WVP is an important index to evaluate the physical properties of films and a vital barrier parameter to prevent water transfer between food and the environment, reflecting the water permeability potential and barrier performance of food-packaging films. In general, low water vapor permeability is preferred because of its high sealing effect and its conduciveness to the long-term storage of food materials [26]. The WVP values of the CS/κC/EEOS composite films are shown in Figure 5.

The WVP value of the CS/κC film was 1.08 g·mm/m^2^·d·kPa, and the WVP values of the composite films increased with the increase in EEOS from 1% to 5%. It can be seen that the WVP values of the films did not change significantly with the addition of a small amount of EEOS (*p* > 0.05). Still, when the EEOS addition level reached 5%, the WVP values of the composite films increased significantly (*p* < 0.05) and reached 1.71 g·mm/m^2^·d·kPa. This indicated that the addition of EEOS affected the physical properties of the CS/κC films, and a higher content of EEOS (5%) led to a significant increase in WVP value (*p* < 0.05). This may be because the excessive anthocyanins in EEOS damage the dense molecular structures of the films, leading to uneven dispersion of the film matrix and a small number of insoluble particles that make the film surface porous [26]. Moreover, plasticization effects increase the molecular mobility and diffusion rates of water vapor through film matrices, which increases WVP [34]. Similar studies have also confirmed that the addition of plant extracts can improve the WVP values of starch-based films [27,34,35]. Tavares et al.’s experiments showed that the addition of a CMC polymer to a maize starch film reduced the WVP values. Their results indicated that the formation of CMC polymeric blends with starch improved water resistance to some extent [35]. This was similar to a study on cassava starch films mixed with Chinese bayberry anthocyanins [27]. Excessive extract formed aggregates and produced more free volumes in the film network to facilitate moisture transfer.

### 3.7. DSC

**Figure 6:**
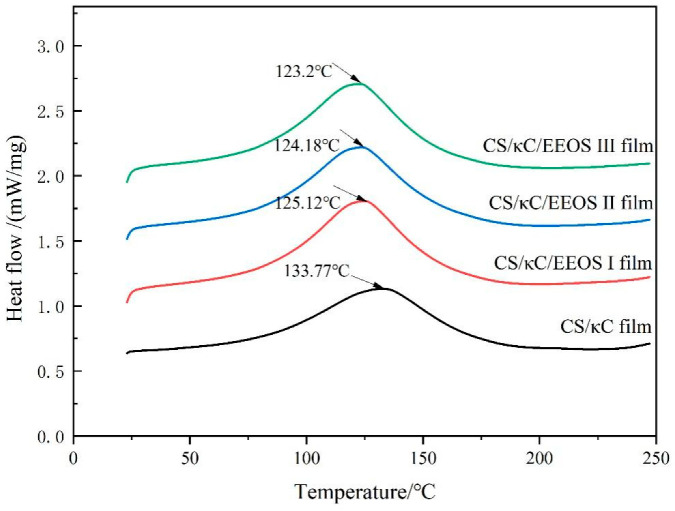
Thermodynamic properties of the CS/κC, CS/кC/EEOS I, CS/кC/EEOS II, and CS/кC/EEOS III composite films.

DSC is a technique to study the interactions between polymer molecules. *Tg* appears as an endothermic shift. The peak should be a first-order phase transition. It can be used to analyze the compatibility and thermal stability of blends. It reflects the molecular movements of various types of polymers and has a great relationship with the kinds of materials and structural properties of composite films [36]. A differential scanning calorimetry analysis was performed on the CS/κC/EEOS composite films. A DSC diagram of the composite films is shown in Figure 6.

The endothermic peak showed that the *Tg* of the CS/κC film, the CS/κC/EEOS Ⅰ film, the CS/κC/EEOS Ⅱ film, and the CS/κC/EEOS Ⅲ film were 133.77 °C, 125.12 °C, 124.18 °C, and 123.20 °C, respectively. Both the CS/κC film and the composite films had peaks between 20 °C and 250 °C, which indicated that the compounds in the composite films had good compatibility. The glass-transition temperatures of the composite films added with EEOS were lower than that of CS/κC, and the glass-transition temperatures of the composite films decreased with the increase in EEOS. Similar results were observed in the study of Wang et al. [37]. The decrease in the glass-transition temperature may be due to the damage of starch chain interaction caused by extract of onion skin and the increase in chain flexibility.

### 3.8. TPC

**Figure 7:**
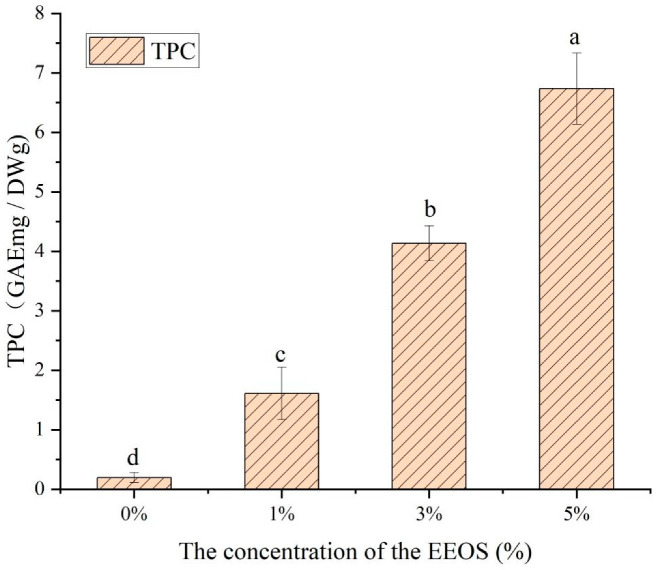
Total phenol contents of the CS/κC, CS/кC/EEOS I, CS/кC/EEOS II, and CS/кC/EEOS III composite films. ^a–d^ Values are given as means ± standard deviation. Different letters in the same line indicate significant difference (*p* < 0.05).

It is well-explained that naturally extracted polyphenolic compounds are excellent antioxidants. Therefore, there is a close relationship between the phenolic contents and the antioxidant activity [30]. The TPC assay determines the amounts of total phenolic compounds in the samples, which are responsible for the antioxidant activity [38]. Therefore, the polyphenol content can be used as an essential indicator for evaluating the antioxidant capacity. Figure 7 shows the total phenolic contents of the CS/κC/EEOS composite films with different amounts of EEOS addition.

The CS/κC composite film itself had a very low TPC content. When EEOS was added to the CS/κC composite films, the total phenolic content of the composite films increased significantly with EEOS concentration. The total phenol content increased from 1.61 GAEmg/DWg to 6.73 GAEmg/DWg with the rise in EEOS from 1% to 5%. The TPC (6.73 GAEmg/DWg) content of the CS/κC/EEOS Ⅲ composite film with 5% EEOS content was the highest. Purple onion skin is rich in anthocyanins and other phenolic compounds, and the content of polyphenols is closely related to antioxidant activity, which significantly increased the total phenolic contents of the composite films, thus improving the biological activity of the composite films. It can also be interpreted that anthocyanin contains phenolic compounds that can provide hydrogen atoms [39]. The higher the concentration of EEOS, the more anthocyanins it has, the more phenolic compounds it has, and the more potent its antioxidant capacity.

### 3.9. Analysis of Antioxidant Properties In Vitro

**Figure 8:**
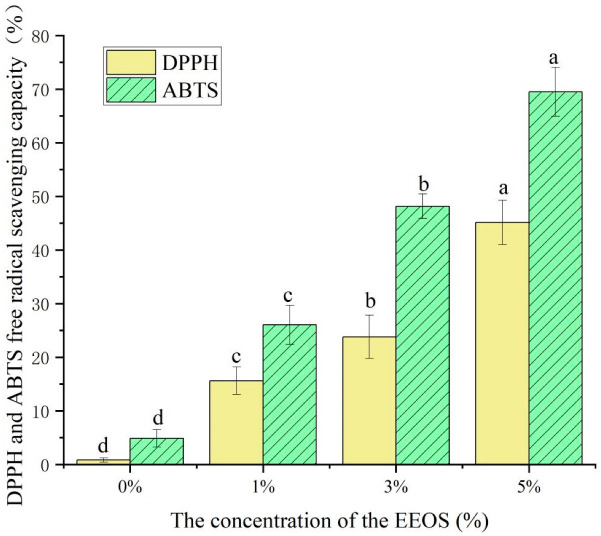
Scavenging activities of the CS/κC, CS/кC/EEOS I, CS/кC/EEOS II, and CS/кC/EEOS III composite films on DPPH and ABTS radicals. ^a–d^ Values are given as means ± standard deviation. Different letters in the same line indicate significant difference (*p* < 0.05).

Antioxidant capacity is essential for active food packaging because free radicals can cause food spoilage and nutrient loss [33]. The in vitro antioxidant capacity of the CS/κC/EEOS composite films is shown in Figure 8.

The single CS/κC film without EEOS had weak scavenging ability for DPPH and ABTS free radicals. With the increase in the EEOS content, the free-radical-scavenging ability of the composite films also gradually improved. When the EEOS additive level was 5%, the DPPH free-radical-scavenging rate reached 45.14%. The ABTS radical-scavenging ability was up to 69.50%. The content of EEOS in the composite films was directly proportional to the free-radical-scavenging activity, which indicated that the anthocyanin content in EEOS had a significant influence on the antioxidant capacity of the composite films. The free-radical-scavenging ability of the CS/κC/EEOS composite films was significantly enhanced with EEOS, which was mainly attributed to active components, such as phenolic compounds and secondary metabolites, that can inhibit oxidation in plants [40]. The increase in free-radical-scavenging activity was also found in the purple cabbage component of anthocyanin-and-biopolymer-based composite films [41]. The polyphenols in EEOS are mainly anthocyanin. The anthocyanins that are rich in EEOS could provide phenolic hydroxyl to capture free radicals, which made up for the deficiency in the antioxidant activity of the CS/κC composite films and improved the antioxidant activity of the films. These results suggested that the presence of the polyphenols in EEOS and the presence of the phenolic compounds in plant extracts contributed to enhancing the free-radical-scavenging potential, thereby enhancing the antioxidant activity. The CS/κC/EEOS composite films containing EEOS had a high antioxidant capacity and could prevent lipid oxidation in food. Therefore, the composite films made in this study can be used as an active packaging material to delay lipid oxidation in foods with high fat content [14].

### 3.10. Analysis of Oxidation Resistance of the Film

**Figure 9:**
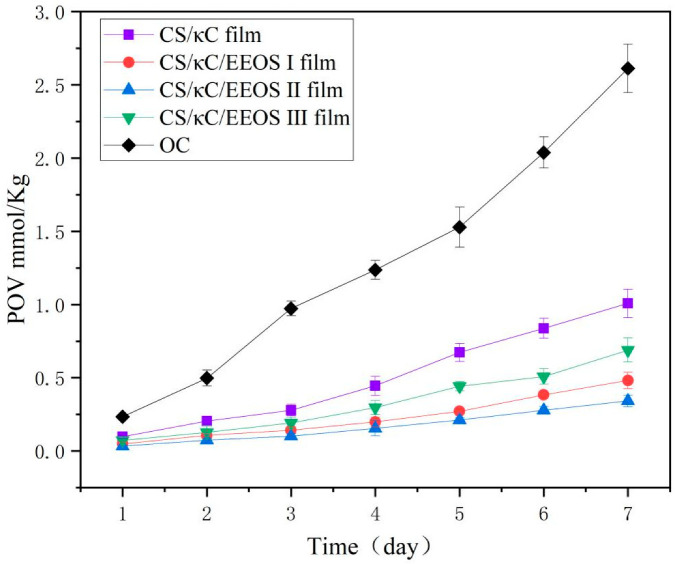
POV values of lard coated with CS/κC/EEOS composite films with different EEOS additive levels.

Good oxidation resistance is significant for active food-packaging film. Therefore, we chose lard as the experimental material to evaluate the antioxidant capacity of the composite films. The POV values representing the oil oxidation performances of the CS/κC/EEOS composite films are shown in Figure 9, and the POV values are proportional to the oxidation degree.

The oxidation degree of unwrapped lard increased from 0.24 to 2.61 g/100 g within 7 days. The POV value of lard decreased obviously after being parceled by the composite films, which indicated that the composite films had a blocking effect on oxygen, reduced the contact between the sample and oxygen, and inhibited the oxidation degree of lard. The composite films without EEOS had poor inhibition effects on lard oxidation. On the 7th day, the POV value of lard reached 1.01 g/100 g, which was higher than that of the composite films with EEOS, indicating that the addition of EEOS improved the ability of the composite films to inhibit oil oxidation and played a certain synergistic role. The addition of EEOS significantly improved the antioxidant activity of the CS/κC films, which was related to the strong free-radical-scavenging ability of the EEOS, which was rich in anthocyanins [38]. Gao et al. [22] also reached a similar conclusion that the POV values of lard wrapped in CS/CA/EBSC composite films were significantly higher than those in CS/CA films. With the increase in the EEOS additive level, the EEOS composite films had a better inhibiting effect on lard oxidation, but the 5% EEOS composite films had a poor inhibiting effect on lard oxidation. This shows that it was not the higher concentration of EEOS, the better the antioxidant effect of the composite film. Excessive EEOS affected the structure of the films, increased the oxygen penetration of the films, and led to lard oxidation. This may be because, with the increase in the amount of EEOS, the microstructures and compactness of the composite films were affected, which weakened the ability of the films to block oxygen. However, the POV value of the lard wrapped with 3% EEOS was 0.34 g/100 g on the 7th day, indicating that the composite films at this concentration had the best inhibition effect on lard oxidation. Similarly, Nilsuwan et al. found that, when epigallocatechin gallate (EGCG) was added to a fish gelatin membrane, it could migrate to high-fat food mimics [42]. The results showed that composite films of biodegradable films and natural active substances have potential application value in food packaging.

### 3.11. Bacteriostatic Analysis

The size of the bacteriostatic rings of *Staphylococcus aureus* (Gram-positive) and *Escherichia coli* (Gram-negative) can be used to evaluate the bacteriostatic activity of composite film samples. The bacteriostatic ring diameters of the CS/κC/EEOS composite films against *S. aureus* and *E. coli* are shown in Table 2.

The inhibition effect of the CS/κC composite films on *S. aureus* and *E. coli* was not pronounced. When EEOS was added to the composite films, the antibacterial effect of the films was improved with the increase in the amount of EEOS. The diameter of the antibacterial ring was gradually increased with the rise in the amount of EEOS. Koosha et al. [43] added black carrot anthocyanin to chitosan/PVA films and found that the antibacterial performance of the chitosan/PVA composite films increased with the increase in black carrot anthocyanin content. The antibacterial ring diameter of the composite films against *S. aureus* was more extensive than that of *E. coli*, which indicated that the antibacterial effect of the composite films against Gram-positive bacteria was better than that against Gram-negative bacteria. Previous studies have reported similar results, suggesting that plant extracts are more effective in killing *S. aureus* than *E. coli* [7]. The flavonoid compounds contained in EEOS can reduce the fluidity of the cell membrane or perforate the membrane through the hydrogen peroxide pathway, thus damaging the cell membranes of microorganisms, leading to the secretion disorder of microbial cells and, thus, inhibiting the growth of microorganisms. Previously, starch-based films with sappan and cinnamon plant extracts showed antimicrobial efficiency that effectively extended the shelf-life of packaged meat [44]. A study showed that the phenolic compounds in EEOS can not only increase the permeability of the cell membrane but can also interfere with the synthesis of microbial genetic material, thus inhibiting the growth of microorganisms and improving the bacteriostatic performances of CS/κC composite films [45]. Liu et al. also reached a similar conclusion when studying the inhibitory effect of CS/polyvinyl alcohol/graphene oxide nanofibrous films loaded with allicin on three foodborne pathogens [46].

## 4. Conclusions

In this study, CS/κC/EEOS composite films were prepared by blending 0%~5% ethanol extract of onion skin with corn starch/κ-carrageenan films, and the effects of EEOS concentration on the physicochemical properties and biological activities of the composite films were discussed. FTIR, XRD, and SEM analyses showed that the addition of EEOS could inhibit the recrystallization of the starch to a certain extent. The microstructures of the composite films were affected, and the surfaces and cross-sections of the films became rough. When the EEOS concentration was 5%, the film structure was coarsest. The addition of EEOS reduced the brightness, increased the opacity and deepened the film color. The mechanical properties of the film were affected: the tensile strength decreased and the elongation at the break increased; the WVP value increased; and DSC showed that the glass-transition temperatures of the composite films were decreased by adding EEOS. In terms of biological activity, the total phenolic content, as well as the DPPH and ABTS free-radical-scavenging rates, increased significantly with EEOS concentration. When the EEOS concentration was 5%, the free-radical-scavenging ability was the highest. The results showed that the composite films with an EEOS concentration of 3% had the best antioxidant effects, and the POV value of lard wrapped with EEOS was the lowest on the 7th day. The inhibitory effect of the films on *S. aureus* was higher than that of *E. coli*, and the diameter of the inhibitory ring increased with the increase in EEOS concentration. From what was discussed above, the optimal addition amount of EEOS was 3%, and that film had the best all-around performance. The prepared composite film has potential application value in food packaging. The results can provide a reference for the production and application of CS/κC/EEOS composite films.

## Figures and Tables

**Table 1:** Color parameters, opaqueness, and pictures of different EEOS concentration composite films.

Film Sample	a *	b *	L *	Opaqueness s/%	Picture
CS/кC film	−1.22 ± 0.04 ^d^	−0.18 ± 0.08 ^d^	90.24 ± 0.33 ^a^	1.06 ± 0.13 ^d^	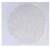
CS/кC/EEOS Ⅰ film	5.40 ± 0.25 ^c^	10.62 ± 0.36 ^c^	78.34 ± 0.39 ^b^	3.55 ± 0.28 ^c^	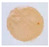
CS/кC/EEOS Ⅱ film	12.68 ± 0.41 ^b^	16.24 ± 0.54 ^b^	66.40 ± 0.87 ^c^	4.74 ± 0.07 ^b^	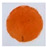
CS/кC/EEOS Ⅲ film	17.26 ± 0.23 ^a^	18.06 ± 0.85 ^a^	59.88 ± 0.91 ^d^	5.56 ± 0.04 ^a^	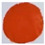

^a–d^ Values are given as means ± standard deviation. Different letters in the same line indicate significant difference (*p* < 0.05).

**Table 2:** Diameter of bacteriostasis circles of CS/κC and CS/κC/EEOS composite films against *Escherichia coli* and *Staphylococcus aureus*.

Extract Concentration	Diameter of the Bacteriostatic Circle (mm)	
	*Escherichia coli*	*Staphylococcus aureus*
CS/кC film	7.06 ± 0.03 ^d^	7.02 ± 0.05 ^d^
CS/кC/EEOS Ⅰ film	11.20 ± 0.14 ^c^	11.48 ± 0.25 ^c^
CS/кC/EEOS Ⅱ film	12.63 ± 0.36 ^b^	14.52 ± 0.22 ^b^
CS/кC/EEOS III film	14.12 ± 0.32 ^a^	15.40 ± 0.38 ^a^

^a–d^ Values are given as means ± standard deviation. Different lowercase letters in the same column indicate significant difference (*p* < 0.05).

## Data Availability

The datasets generated and analyzed during the current study are available from the corresponding author on reasonable request.
